# Nanomaterials Exhibiting Enzyme-Like Properties (Nanozymes): Current Advances and Future Perspectives

**DOI:** 10.3389/fchem.2019.00046

**Published:** 2019-02-05

**Authors:** Sanjay Singh

**Affiliations:** Division of Biological and Life Sciences, School of Arts and Sciences, Ahmedabad University, Ahmedabad, India

**Keywords:** nanozymes, peroxidase, oxidase, superoxide dismutase, metalloenzymes

## Abstract

Biological enzymes are macromolecular catalysts that catalyze the biochemical reactions of the natural systems. Although each enzyme performs a particular function, however, holds several drawbacks, which limits its utilization in broad-spectrum applications. Natural enzymes require strict physiological conditions for performing catalytic functions. Their limited stability in harsh environmental conditions, the high cost of synthesis, isolation, and purification are some of the significant drawbacks. Therefore, as an alternative to natural enzymes, recently several strategies have been developed including the synthesis of molecules, complexes, and nanoparticles mimicking their intrinsic catalytic properties. Nanoparticles exhibiting the properties of an enzyme are termed as “nanozymes.” Nanozymes offer several advantages over natural enzymes, therefore, a rapid expansion of the development of artificial biocatalysts. These advantages include simple methods of synthesis, low cost, high stability, robust catalytic performance, and smooth surface modification of nanomaterials. In this context, nanozymes are tremendously being explored to establish a wide range of applications in biosensing, immunoassays, disease diagnosis and therapy, theranostics, cell/tissue growth, protection from oxidative stress, and removal of pollutants. Considering the importance of nanozymes, this article has been designed to comprehensively discuss the different enzyme-like properties, such as peroxidase, catalase, superoxide dismutase, and oxidase, exhibited by various nanoparticles.

## Introduction

Recent expansions in the area of nanotechnology have led to an exponential growth in development of nanomaterials exhibiting natural enzyme-like activities (Nanozymes), possessing several advantageous merits (Wei and Wang, 2013). Natural enzymes require strict physiological conditions for performing catalytic functions. Their limited stability in harsh environmental conditions, the high cost of synthesis, isolation, and purification are some of the significant drawbacks. Unlike natural enzymes, nanozymes offer unflinching biocatalytic activity even in the extreme conditions of pH, temperatures and resistance to the digestion from proteases. Therefore, it was imperative to develop efficient alternatives for artificial enzymes. Thus, in recent years, researchers have focused on utilizing the catalytic powers of chemical molecules, such as cyclodextrins, metal-complexes, porphyrins, polymeric, and supramolecules, as alternatives to natural enzymes (Jeon et al., 1998; Raynal et al., 2014). However, the catalytic efficiency and biocompatibility were some of the harboring concerns with these molecules.

In this pursuit, the discovery of iron-oxide-based artificial peroxidase enzyme was reported by Gao et al. (2007). It was the first report to show that, similar to natural horseradish peroxidase enzyme, the inorganic nanoparticles could also exhibit the oxidation of typical peroxidase substrates and could be used for antibody-based identification, separation, and detection of analytes of interest. Subsequently, several nanomaterials (metallic, metal oxides, and carbon-based nanoparticles) were investigated for possessing intrinsic biological enzyme-like catalytic activities, predominantly, catalytic activities similar to peroxidase, oxidase, catalase, and superoxide dismutase enzymes (Karakoti et al., 2010; Pirmohamed et al., 2010; Singh, 2016; Karim et al., 2018; Zhang et al., 2018; Zhao et al., 2018). This rapid expansion in nanozyme research was possible due to the several advantages inorganic nanoparticles possess over natural enzymes, such as simple methods of synthesis, low cost, high stability, robust catalytic performance, and smooth surface modification. Therefore, nanozymes are being widely explored to establish a wide range of applications in biosensing, immunoassays, disease diagnosis and therapy, theranostics, cell/tissue growth and proliferation, protection from oxidative stress, and removal of pollutants (Xie et al., 2012; Lin et al., 2014; Zhou et al., 2017). Broadly, the nanozymes that have been discovered so far can be divided into two categories, antioxidants and pro-oxidants, considering their functions as either scavenging the free radicals or generating free radicals during the catalytic reaction, respectively.

## Antioxidant Nanozymes

In the biological system, antioxidants are required to protect the cells/tissues from damage imposed by the excess of free radicals, generated during the normal biochemical reactions of the body. The human body has a well-established endogenous antioxidant system, which is mostly orchestrated by free radical scavenging enzymes such as catalase, superoxide dismutase (SOD), glutathione peroxidase, glutathione reductase, peroxiredoxins, etc. Among inorganic antioxidants, cerium oxide nanoparticles (CeNPs) are reported by Self and colleagues to exhibit scavenging of superoxide radicals and degradation of hydrogen peroxide under *in vitro, in vivo*, and other animal models (Korsvik et al., 2007; Heckert et al., 2008a; Karakoti et al., 2009).

The below section will comprehensively cover the overview of nanoparticles reported to exhibit antioxidant enzyme-like activities.

### Superoxide Dismutase Mimetic Nanoparticles

**Figure 1:**
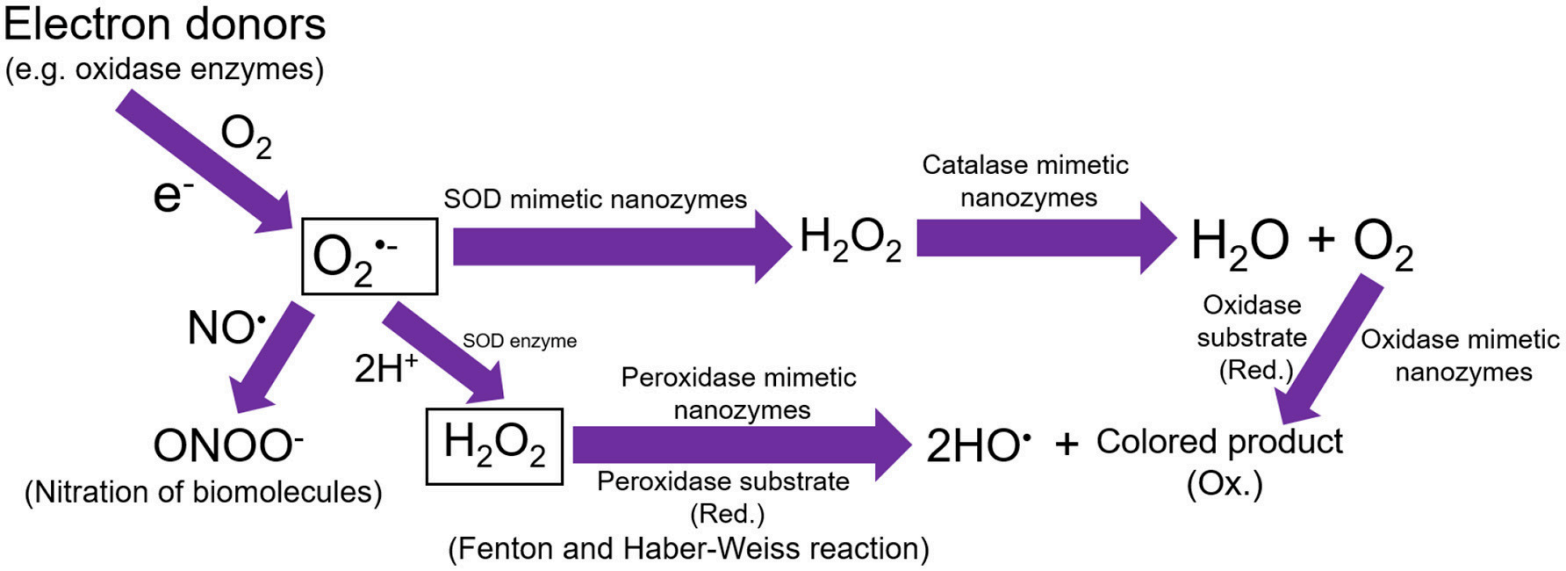
Schematic representation showing that the single electron donors can produce superoxide anions. These superoxide anions could further react with NO or 2H^+^ to produce ONOO^−^ anions and hydrogen peroxide, respectively. The SOD mimetic nanozymes can dismutate the superoxide anions into hydrogen peroxide, which can further be degraded into water and molecular oxygen by catalase mimetic nanozymes. Hydrogen peroxide can also lead to the generation of hydroxyl radicals in presence of peroxidase mimetic nanozymes, however, oxidase mimetic nanozymes can directly oxidize the substrate into colored product without the involvement of hydrogen peroxide.

SOD enzyme is one of the unique antioxidant enzymes, and not many nanoparticle types have been developed to exhibit the superoxide anions scavenging activity, except CeNPs. Therefore, this subsection will mainly focus on the CeNPs and their SOD enzyme-like activities. Our research group has also done comprehensive research on the mechanism of protection of mammalian cells stressed with high superoxide radicals (SOD mimetic activity) and hydrogen peroxide (catalase mimetic activity) (Singh et al., 2016; Patel et al., 2018; Rather et al., 2018). Mechanistically, it has reported with substantial evidence that the high Ce^+3^/Ce^+4^ ratio of surface “Ce” atoms from CeNPs exhibit SOD mimetic activity, whereas, the lower ratio of Ce^+3^/Ce^+4^ leads to the catalase mimetic activity (Dhall and Self, 2018) (Figure 1). Since the natural SOD enzyme plays a vital protective role in the scavenging of superoxide anions, however, its short-term stability and the high cost of synthesis creates an opportunity to develop an efficient alternative. In one such effort, manganese-containing biscyclohexylpyridine complex (M40403), was synthesized, which exhibited SOD enzyme-like activities but only to a certain extent (Muscoli et al., 2003). Inspired by this discovery, Seal and Self groups first reported the use of CeNPs as an alternative to SOD enzyme with better catalytic efficiency (Heckert et al., 2008a; Karakoti et al., 2009, 2010). Through kinetics, authors also state that CeNPs (3–5 nm) showed better SOD mimetic activity than native CuZn SOD (rate constant: 3.6 × 10^9^ M^−1^ s^−1^ and 1.1 × 10^5^ M^−1^ s^−1^, respectively) (Korsvik et al., 2007). When comparing the catalytic efficiency of single CeNP with the most recently calculated rate constant of CuZn SOD (~1.3–2.8 × 10^−9^ M^−1^s^−1^) for their SOD activity, the former showed better scavenging of superoxide radicals than the authentic enzyme itself. The superoxide anion scavenging ability of the CeNPs has also been confirmed by electron paramagnetic resonance (EPR) measurements, and the possible dismutation of superoxide radicals by CeNPs could be catalyzed as follows.

$${\text{O}}_{2}^{•-}+{\text{Ce}}^{+4}\to {\text{O}}_{2}+{\text{Ce}}^{+3}$$

$${\text{O}}_{2}^{•-}+{\text{Ce}}^{+3}+2{\text{H}}^{+}\to {\text{H}}_{2}{\text{O}}_{2}+{\text{Ce}}^{+4}$$

It has also been reported that CeNPs show an auto-regeneration process, whereby nanoparticles regenerate the Ce^+3^ oxidation state atoms from oxidized Ce^+4^ atoms (during the superoxide radical dismutation process), within a few days, which further makes CeNPs ready to neutralize another superoxide radical. Although some initial studies have reported that CeNPs could also scavenge hydroxyl radicals, however, any conclusive data was not shown as their EPR data suggest that CeNPs does not neutralize hydroxyl radicals (Heckert et al., 2008b; Zou et al., 2018). Our group has found that CeNPs have a high affinity for superoxide radicals and therefore eliminate them efficiently. In one of our recent studies, we explored the antioxidant and concomitant anti genotoxic nature of CeNPs toward the oxidative insult generated by buthionine sulfoximine (BSO) in human keratinocytes (HaCaT cells) (Singh et al., 2016). It is reported that BSO inhibits the synthesis of the γ-glutamylcysteinesynthetase enzyme, which leads to the depletion of glutathione (GSH), thus negatively modulates the cellular redox potential. Our results suggested that CeNPs can protect HaCaT cells from BSO-induced oxidative damage when cells were pre-incubated with CeNPs. We estimated the cell survival and intracellular levels of ROS, release of lactate dehydrogenase enzyme (due to membrane damage), and nuclear fragmentation.

Further, the study of the expression of antioxidant genes and proteins, [thioredoxin reductase (TrxR) and peroxiredoxin 6 (Prx6)] showed that, due to pretreatment of CeNPs, there was limited need for the induction of these antioxidant genes and concomitant enzymes involved in the defense against oxidative stress. Although CeNPs are the most studied and well-established nanozyme exhibiting SOD enzyme-like activity, Platinum nanoparticles (PtNPs) encapsulated in apo-ferritin have also been shown to exhibit SOD enzyme-like activities (Jawaid et al., 2014; Liu et al., 2014). PtNPs retain their SOD mimetic activity in cell culture models; however, the overall efficiency was reported to be significantly lower than CeNPs (on the weight basis). Growing evidence reports several applications of CeNPs toward the protection of cell culture and animal models from free radicals, and concurrently argues for the antioxidant role of CeNPs. However, many of these reports do not confirm the type of activity (either SOD or catalase) of CeNPs, which could be due to the poor characterization. Dugan and co-workers (Ali et al., 2004, 2008) have shown that fullerenes (C60) and their derivatives can also exhibit the SOD enzyme-like activities. Using EPR studies, they found that fullerenes could also scavenge the superoxide anions as well as hydroxyl radicals with almost the same efficiency. Exposure of these fullerenes to *in vitro* cultured cortical neurons imparted protection against the toxic effects induced by N-methyl D-aspartate. Fullerenes protected the Ab-peptide by the scavenging of the superoxide radicals thus the neurotoxicity was also significantly reduced. Authors later reported a tris-malonic acid derivative of the fullerene molecule that has lower efficiency than natural SOD enzyme, with a comparable rate constant of [k_(fullerene)_] of 2 × 10^6^ mol^−1^ s^−1^], about 100-fold slower than the SOD enzyme (Ali et al., 2004).

### Catalase Mimetic Nanoparticles

Biological catalase enzyme catalyzes the decomposition of the excess of cellular hydrogen peroxide into water and molecular oxygen. Generally, the dismutation of superoxide radicals by SOD enzyme leads to the generation of hydrogen peroxide. Owing to the significant role of hydrogen peroxide toward either biological signaling or production of extremely reactive hydroxyl radicals, it is a stable and less reactive species in the cytoplasm. It is well-established that hydrogen peroxide undergoes “Fenton reaction” in the presence of any transition metal ions and forms hydroxyl radicals, which are detrimental to biological molecules [(Heckert et al., 2008b; Leifeld et al., 2018)]. Therefore, it is essential that the excess of cytoplasmic hydrogen peroxides must be converted to water and molecular oxygen using catalase enzyme. However, in the absence of functional catalase enzyme, the excess of hydrogen peroxides could give rise to several diseases, such as acatalasemia, diabetes, and vitiligo. Therefore, an alternative to biological catalase is imperative, and researchers have developed several types of nanoparticles exhibiting catalase enzyme-like activities including cerium oxide, iron oxides, gold nanoparticles (AuNPs), and Cobalt oxide nanoparticles (Mu et al., 2014; Wang et al., 2016; Zhang et al., 2017; Bhagat et al., 2018; Vallabani and Singh, 2018). Among several types of nanomaterials reported, CeNPs (high Ce^+4^/^+3^ ratio), and iron oxide nanoparticles have been studied in detail. Recently, we have investigated the alteration in catalase mimetic activity of CeNPs when suspended in biologically relevant buffers, and our results show that unlike SOD mimetic CeNPs (high Ce^+3^/^+4^ oxidation state), catalase mimetic CeNPs (high Ce^+4^/^+3^ oxidation state) are robust and do not compromise their catalytic activity (Singh and Singh, 2015). The degradation of hydrogen peroxide by CeNPs can be represented as follows:

$${\text{2H}}_{\text{2}}{\text{O}}_{\text{2}}{\text{+Ce}}^{\text{+4}}\to {\text{2H}}_{\text{2}}{\text{+2O}}_{\text{2}}{\text{+Ce}}^{\text{+3}}$$

Our recent study suggests that catalase mimetic CeNPs can protect hepatic cells from cytotoxicity and genetic damage induced from the elevated concentrations of hydrogen peroxide in the absence of functional cellular catalase enzyme. Human hepatic cells were exposed with 3-aminotriazole (3-AT) to artificially inhibit the function of cellular catalase enzyme, which resulted in the high level of hydrogen peroxide accumulation. Results reveal that CeNPs can protect hepatic cells from 3-AT mediated early apoptosis and DNA damage (Singh and Singh, 2019). The genomics and proteomics studies revealed that CeNPs did not elicit the natural antioxidant defense system of the hepatic cells even in the absence of functional catalase enzyme, which suggested that the cellular protection was solely due to the hydrogen peroxide degradation by catalase mimetic CeNPs. This finding demonstrates the reinforcement of CeNPs as pharmacological agents for the treatment of diseases related to nonfunctional biological catalase enzyme in the mammalian cells.

Additionally, there are few hydrolytic nanozymes reported to hydrolyze the toxic biological agents such as neurotoxic organophosphates. In an attempt, Khulbe et al. (2018) showed the development of Zr-incorporated CeO_2_ nanocatalyst for efficient hydrolysis of nerve agents such as methyl paraoxon to less toxic monoesters. It was a first report showing a nanozymes catalyzing a two-step hydrolysis reaction with a faster catalytic rate (t_1/2_ value of 1.2 and 3.5 min for methyl paraoxon and methyl parathion hydrolysis, respectively) than the single-step hydrolysis reaction reported earlier by others. Thiol passivated AuNPs have also been found to exhibit hydrolytic enzyme-like activities by following the hydrolytic cleavage of phosphate diester bonds from DNA. Cleavage of plasmid DNA by AuNPs resulted in the conversion of the compact supercoiled conformation of the plasmid (form I) to the relaxed circular (form II), due to a cut in one of the two strands leading to a remarkable change in mobility of the nicked plasmid during electrophoresis (Mancin et al., 2016).

Iron oxide nanoparticles are also reported to exhibit catalase enzyme-like activities, however, strong supporting literature has not been developed so far. Chen et al. have recently reported that iron oxide nanoparticles show a pH (7.4) dependent nanozymatic activity (Chen et al., 2012), however, at acidic pH peroxidase enzyme-like activity was reported. Using EPR studies, authors confirmed that hydroxyl radical formation occurs at acidic pH but not at neutral pH, suggesting that at acidic conditions iron oxide nanoparticles show “Fenton-like chemistry.” This observation was further supported by their cellular toxicity studies, where iron oxides entrapped in acidic lysosomes undergo hydroxyl radical formation and thus induce cell death, however, iron oxide nanoparticles dispersed in cytoplasm did not cause any harm to cells. The latter effect could be due to the catalase-like activity of iron oxide nanoparticles in neutral pH cytoplasm.

Although there have been several such studies with a variety of nanoparticles showing biological catalase enzyme-like activities, however, limited to the *in vitro* studies. Further validation into higher order *in vivo* experimental models is imperative in order to explore the potentials of antioxidant nanoparticles. Further, detailed elucidation of the mechanism of antioxidant activity of nanozymes in biological systems would assist their broad applications in biomedicine.

## Pro—oxidant Nanozymes

The term “pro-oxidant nanozymes” refers to the action of nanozymes which induces oxidative stress by producing free radicals in mammalian cells or inhibiting their antioxidant system. Common drugs such as analgesic paracetamol and anticancerous methotrexate are known to generate free radicals and therefore considered as pro-oxidants. Similarly, transition metals such as Iron and Copper etc. are also reported to undergo Fenton reaction and Haber-Weiss reaction, and subsequently produce excessive free radicals (Rahal et al., 2014). Therefore, nanozymes catalyzing the reactions (such as peroxidase and oxidase), which involves the generation of free radicals, can also be regarded as pro-oxidant nanozymes.

### Peroxidase Mimetic Nanoparticles

Natural peroxidases consist of a large family, and they predominantly utilize hydrogen peroxide to oxidize peroxidase substrates. Peroxidase enzymes are of considerable importance because they act as detoxifying agents for free radicals (e.g., glutathione peroxidase) and also facilitate the defense against invading pathogens (e.g., myeloperoxidase) (Strzepa et al., 2017). Further, HRP is well known for their applications in bioanalytical and clinical chemistry, for the conversion of colorless substrate into colored product leading to the detection of analytes. We and others have recently shown that specific nanomaterials can exhibit peroxidase enzyme like catalytic activities. A schematic representation of peroxidase activity exhibited by nanozymes has been shown in Figure 1. Although iron oxides are predominantly reported to have excellent peroxidase enzyme-like activity, other nanomaterials have also received considerable attention. The very first report by Gao et al. showed that different sizes of iron oxide nanoparticles (30, 50, and 300 nm) could oxidize the colorless TMB into a blue colored product in the presence of hydrogen peroxide at acidic pH (Gao et al., 2007). However, the smaller sized particles could exhibit higher peroxidase-like activity than corresponding bigger sized ones. Authors compared the peroxidase activity of iron oxide nanoparticles with natural HRP enzyme and found that in both cases it was dependent on the reaction temperature and pH. However, unlike HRP, the nanoparticles remain stable and retain their catalytic activity after the incubation at a broader range temperature [4–90°C and pH (1–12)]. The kinetic analysis revealed that the substrate affinity (Km) value of iron oxide nanoparticles with hydrogen peroxide was higher than HRP (154 and 3.7 mM, respectively), suggesting that a higher concentration of hydrogen peroxide is needed to obtain the maximum activity for iron oxide nanoparticles.

Further, the Km value of iron oxide nanoparticles with TMB was about four times lesser than HRP (0.098 and 0.43 mM, respectively), suggesting that nanoparticles have a higher affinity for the substrate (TMB) than HRP, therefore, at the same molar concentrations, nanoparticles showed 40 times higher activity than HRP. Soon after this work, several reports have been published on the peroxidase activity and related sensing and detection applications of iron oxide and other nanoparticles. Among them, Wei and Wang developed a unique sensing platform for the detection of hydrogen peroxide and glucose using iron oxide nanoparticles as a peroxidase mimic (Wei and Wang, 2008). The results of these studies stimulated rapid expansion in the use of iron oxide nanoparticles as an alternative of peroxidase enzyme and researchers across the world use them for different applications. Among iron oxide nanoparticle types, magnetite nanoparticles have grabbed most attention and thus been studied extensively. There are several more types of iron-based nanomaterials which are reported to exhibit peroxidase-like activity. Among them, Fe-S nanosheets were prepared by a micelle-assisted strategy and their peroxidase activity was studied. It was argued that due to the large surface area of nanosheets, the peroxidase activity was found to be better than that of corresponding spherical nanoparticles of Fe-S (Dai et al., 2009). Similarly, Fe-S nanoneedles are also shown to have better peroxidase activity than spherical Fe-S nanoparticles (Dutta et al., 2012).

Additionally, FeTe nanorods demonstrated better peroxidase activity than spherical iron oxide nanoparticles. Thus, these studies suggest that the shape and size of nanoparticles significantly governs the peroxidase activity of nanozymes. Other nanomaterials such as nanostructured layered double hydroxide (LDH) and CuS superstructures are also reported to exhibit excellent peroxidase-like activity, which has been translated into constructing electrochemical and colorimetric sensors (He et al., 2012; Zhang et al., 2012). The peroxidase mimetic activity exhibited by nanozymes could be catalyzed by following two steps:

$$3{\text{H}}_{2}{\text{O}}_{2+}\text{Peroxidase mimetic nanozymes}\to {\text{6HO}}^{•}$$

$$\begin{array}{l} 6{\text{HO}}^{•}+\text{2Peroxidase substrate}{\left(\text{TMB}/\text{OPD}/\text{ABTS}\right)}_{\text{Red.}}\to \\ \;2{\left(\text{TMB}/\text{OPD}/\text{ABTS}\right)}_{\text{Ox.}}+6{\text{H}}_{2}\text{O} \end{array}$$

All of the studies reporting intrinsic peroxidase-like activity of iron oxide nanoparticles have shown that acidic pH (pH 4.0) is one of the fundamental requirements driving the oxidation of peroxidase substrates (TMB, OPD, ABTS), which finally results in corresponding colorimetric product formation used in the detection of a variety of analytes. Therefore, the detection sensitivity is significantly dependent on the ability of oxidation of peroxidase substrate by iron oxide nanoparticles in the presence of hydrogen peroxide. The limited sensitivity and pH condition constraint is a major limitation with the sensing of biomolecules at physiological pH. In this context, we have recently developed a strategy which can avoid the fundamental limitation of acidic pH of peroxidase reaction and shift the optimum pH for peroxidase activity of iron oxide nanoparticles at physiological pH by using ATP (Vallabani et al., 2017). We found that in the presence of ATP, iron oxide nanoparticles exhibit strong peroxidase activity at physiological pH. It was clear that ATP facilitates the single electron transfer reaction, through complexation with iron oxide nanoparticles, which leads to the generation of hydroxyl radicals responsible for enhanced peroxidase activity at physiological pH. Iron oxide nanoparticles showed higher affinity to TMB (Km = 0.37 mM) at pH 7.4 than at pH 4.0 (Km = 0.43 mM). Nanoparticles also showed higher affinity to hydrogen peroxide (Km = 54.6 mM) than HRP (Km = 3.7 mM), whereas higher reaction velocity (4.83 times) than HRP. We also utilized this strategy to develop a single step detection of glucose with a detection limit of 50 μM. This method was further extended to monitor glucose levels in human blood serum within 5 min at pH 7.4. AuNPs are also reported to exhibit peroxidase-like activity, which has been used for the detection of several biomolecules. Our research group has shown that the peroxidase-like activity of AuNPs (30 nm) can be improved by at least three-fold in the presence of ATP (Shah et al., 2015). Mechanistically, we found that negatively charged ATP facilitates to stabilize positively charged, oxidized TMB through a simple electrostatic interaction. A similar observation has also reported with the use of ionic liquids, which are high viscosity liquids, to improve the thermal stability of oxidized products but completely inhibit the enzyme-like activity of nanoparticle (Lin et al., 2013). Therefore, ATP can be used for selectively boosting the peroxidase-like activity of nanomaterials, which can subsequently be translated into the sensitive detection of analytes. V_2_O_5_ nanowires are also reported to exhibit intrinsic peroxidase activity, following the similar catalytic reactions as described above. André et al. (2011) have recently reported V_2_O_5_ nanowires showing an exceptional peroxidase reaction with a turnover frequency (k_cat_) of 2.5 × 10^3^ s^−1^. The reported Km values of the nanowires for the oxidation of ABTS and hydrogen peroxide was found to be 0.4 and 2.9 μM, respectively at pH 4.0. These values are significantly smaller than the reported kinetic values of HRP reported earlier. Another report by Natalio et al. shows that the peroxidase activity of V_2_O_5_ nanozymes could be used for a potential alternative to conventional anti-biofouling agents to avoid marine biofouling (Natalio et al., 2012). Several other types of bimetallic and composite nanomaterials are reported to show excellent peroxidase enzyme-like activity, thus illustrate the growing interest and efforts for developing novel nanozymes to efficiently catalyze the biological reactions.

### Oxidase Mimetic Nanoparticles

The reactions catalyzed by oxidase enzyme involve oxidation of the substrate by molecular oxygen, which is converted into water or hydrogen peroxide. Unlike the peroxidase reaction, oxidase enzymes do not require hydrogen peroxide, instead they produce H_2_O_2_ and in some cases superoxide radicals. Due to the *in situ* generation of hydrogen peroxide and superoxide radicals, oxidase enzyme and nanozymes imitating this oxidase activity can efficiently oxidize the colorless substrates into corresponding color products, which makes them ideal agents for detection of biological or chemical molecules. Recently several nanomaterials are reported to exhibit oxidase enzyme-like activities (Luo et al., 2010; Cao and Wang, 2011; Fan et al., 2011; He et al., 2011; Wan et al., 2012; Shah et al., 2018). A schematic representation of oxidase activity exhibited by nanozymes has been shown in Figure 1.

It is well documented that the properties of nanoparticles can be tuned by altering their methods of synthesis, surface modification, size, shape, and even composition. Researchers have utilized these strategies to develop materials with different properties and activities, including the nanozymatic activities. In this context, based on the variation in Ce^+3^/^+4^ ratio, CeNPs are reported to show SOD, and catalase-like activities (discussed above), however, coated with dextran showed oxidase mimicking properties (Asati et al., 2009, 2011). Authors have shown that oxidase mimicking CeNPs could oxidize several colorimetric substrates (ABTS, TMB, and DOPA) under acidic pH in the absence of hydrogen peroxide. The oxidase activity of CeNPs was reported to be dependent on pH, size and dextran coating thickness. The reaction kinetics of CeNPs was compared with HRP and a faster rate constant for the nanozyme was observed (1–7 × 10^−7^ M^−1^s^−1^ of CeNPs than 1 × 10^−8^ M^−1^s^−1^ of HRP). Later, Perez and co-workers utilized the oxidase activity of CeNPs for the development of an assay, for the detection of lung tumor cells. Authors conjugated CeNPs with poly (acrylic acid) (PAA) and subsequently functionalized by folic acid. This conjugate was specifically able to recognize lung tumor cells (A549 cell culture model), which selectively express the elevated levels of folate receptors.

In addition to CeNPs, few other nanoparticles have recently been studied for oxidase-like properties. Among them Fe_2_O_3_ nanowires were reported to exhibit oxidase enzyme-like activity, and a glucose sensor was developed by fabricating an array of Fe_2_O_3_ nanowires. This system showed a linear range of glucose detection (0.015–8 mM) with a limit of detection of 6 mM (Cao and Wang, 2011). We have also reported the synthesis of Fe-Pt alloy nanoparticles using non-ionic surfactant polyoxyethylene cholesteryl ether. These alloy NPs exhibited a robust oxidase enzyme-like activity with about 10-folds of the reaction velocity compared to the other oxidase mimicking nanoparticles reported. Analysis of kinetic parameters (Km and Vmax) of Fe-Pt alloy nanoparticles revealed that the Km value for the affinity between the substrate (TMB) and Fe-Pt alloy NPs is 0.03 mM, suggesting that the affinity with the substrate is lower than the other compared Pt-based bi-metallic nanoparticles. However, the reaction velocity (Vmax) was found at 1.42 × 10^−5^ mM/s, ten-folds higher than the most Pt-based catalytic nanoparticles (8.3 × 10^−6^ and 0.26 × 10^−5^ for Au-Pt and Pd-Pt alloy nanoparticles, respectively) (He et al., 2011; Zhang et al., 2011).

A biocompatibility study revealed that these NPs are nontoxic to human liver cells (up to 150 μM), suggesting that they hold strong potential to be used for multiple biomedical applications (Karakoti et al., 2010). Another report showed that MnO_2_ nanowires could also show oxidase enzyme-like activity. These nanowires were further conjugated with antibodies and utilized for development of an immunoassay of sulfate-reducing bacteria. A MnO_2_ incorporated immunoassay platform showed better pathogen detection performance than HRP-based ELISA, although both methods showed good sensitivity and high selectivity toward bacteria (Wan et al., 2012).

Rossi and co-workers reported the oxidase enzyme-like activity in citrate-capped AuNPs by catalyzing the aerobic oxidation of glucose with dissolved oxygen, in a similar reaction catalyzed by an oxidase enzyme (Comotti et al., 2004; Beltrame et al., 2006). This report was surprising as other metallic nanoparticles, such as Ag, Cu, Pt, and Pd, did not show any significant oxidase-like activity. However, authors reported the Eley-Rideal mechanism of catalysis, which supports the hypothesis of glucose being adsorbed on the AuNPs surface followed by reaction with molecular oxygen. This reaction produces gluconic acid and hydrogen peroxide by following the typical Michaelis–Menten reaction kinetics. Through kinetic parameter analysis authors reported that native enzyme was ~55 times more active than the AuNPs-based nanozyme. Later, Fan et al. (2011) developed an innovative microRNA sensing technology utilizing the oxidase mimicking activity of AuNPs. Considering the different affinities of AuNPs for ssDNA and dsDNA, and the coupling of the system with HRP, the colorimetric or chemiluminescent signals were generated, which could offer the detection of single-base-pair mismatch differentiation (Luo et al., 2010; Zheng et al., 2011).

## Summary and Future Prospects

Although it is well established that nanozymes possess several distinct advantages over natural enzymes as well as other reported artificial enzymes, they still face several limitations. These issues need to be addressed to utilize their biomedical potential to the fullest. Nanozymes hold all the physicochemical and optoelectronic properties of nanomaterials including size, shape, and composition-dependent unique properties. The interesting plasmonic properties of noble metal nanoparticles and superparamagnetic properties of iron oxide and other magnetic nanoparticles could be developed into an efficient theranostic system. Recent developments in the novel and easy surface modification strategies of nanoparticles could be used for surface decoration of nanozymes with targeting ligands for identification of the cells/tissues of interest. Efforts have been made to develop such multifunctional nanozymes; however, such materials frequently lose the catalytic effect upon surface modification. It is of prime importance to investigate the possible alteration of nanozymes activity upon dispersing them into biologically relevant buffers. With SOD mimetic CeNPs, we have observed that nanoparticles lose their SOD-like activity when dispersed in phosphate buffer (pH 7.4) (Singh et al., 2011). Mechanistically, it was found that “Ce” has a very high affinity for phosphate anions, producing cerium phosphate, which does not show SOD mimetic activity.

**Figure 2:**
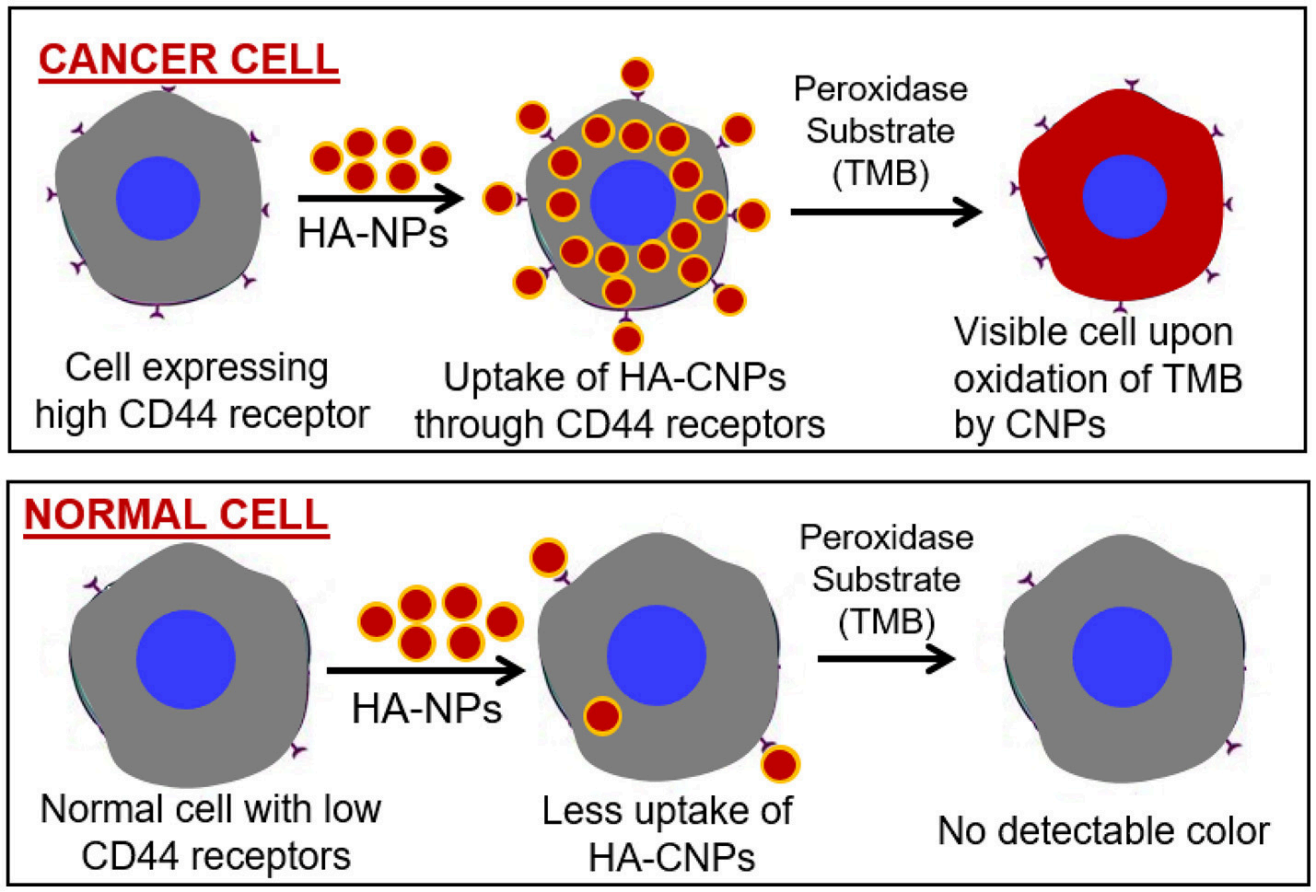
Peroxidase enzyme mimetic nanozymes can differentiate between cancer and normal cells. Peroxidase mimetic cerium oxide nanoparticles (CNPs) coated with HA can bind with the CD44 receptors expresses selectively on cancer cells. During peroxidase reaction these nanoparticles could convert the peroxidase substrate into colored product leading to the identification of cancer cells. Due to the low expression of CD44 receptors on normal cells, peroxidase mimetic nanoparticles would not be attached with cells and thus exhibit no peroxidase reaction.

Similarly, the catalytic activity of other nanozymes must also be investigated when dispersed in relevant buffers, to achieve any biological application. Therefore, strategies which could produce nanozymes coated with desired biomolecules without any significant drop in catalytic activity could be developed for sensing applications. For example, peroxidase activity mimicking nanoparticles, coated with hyaluronic acid (HA), could be used to identify the CD44 overexpressing cancer cells by merely performing a peroxidase reaction (Figure 2).

The catalytic efficiency of most nanozymes is still poorer than natural enzymes and other organic catalysts. Therefore, in the near future, efforts must be devoted toward developing high-performance nanozymes. Some progress has already been made in this direction. We and others have identified few molecules which can efficiently boost the catalytic activity of nanozymes (Simos et al., 2012; Wang et al., 2017; Shah and Singh, 2018). Wang et al. (2017) have shown that the peroxidase-like activity of porous LaNiO_3_ nanocubes was improved by inducing its +3 oxidation state in LaNiO_3_ perovskite. The peroxidase-like activity of porous LaNiO_3_ perovskite with Ni^+3^ was ~58-folds, which was ~22-folds higher than that of NiO with Ni^+2^ and Ni nanoparticles with Ni^0^.

Biological enzymes are highly selective to their targets; however, nanozymes show limited selectivity toward their substrates. For example, most of the nanozymes showing peroxidase-like activity are reported to be used for glucose detection. The activity seen is mainly due to the glucose oxidase enzyme, rather than peroxidase active nanozymes. Therefore, efforts must be devoted to developing nanozymes of high selectivity toward the given substrates. Interestingly, Dhall et al. (2017) have shown that tungstate and molybdate can inhibit the phosphatase activity without altering the oxidative state of CeNPs, however, this did not affect the catalase activity of nanoparticles. These observations suggest that nanozymes do have specific reaction hot-spots on their surface which undergo catalytic reactions. Therefore, it might be possible to inhibit one of the catalytic activities from the nanozymes exhibiting multiple enzyme-like properties. There are metabolic processes orchestrated by multienzyme complexes, which offer several advantages over individual enzyme-catalyzed reactions. However, a functional nanozyme with multiple enzyme-like activities is still limited. Therefore, synthesis of such multifunctional nanozymes would be the hot topic of study in this area. We have reported the synthesis of a multifunctional enzyme consisting of Gold (core)-CeO_2_ (shell) nanoparticles (Au/CeO_2_ CSNPs) exhibiting peroxidase, catalase, and superoxide dismutase enzyme-like activities. The nanozyme activities could be tuned simply by varying the reaction pH.

Further, the kinetic parameters of peroxidase reaction shown by nanozyme were comparable to natural HRP enzyme. Additionally, the functional assemblies of several individual nanozymes together would also open new paradigms for development of nanozymes with synergistic catalytic activity of different components (Wilner et al., 2009). More of these developments would open new directions for the development of single platform sensors and theranostics, which could be applicable in multiple biosensing and biomedical applications. Most of the nanozymes are reported to exhibit their catalytic activity by redox activity by surface atoms. However, the catalytic activity may be further improved by manipulating the core of the nanozymes by doping with some rare-earth elements. Such strategies would add more redox “hot-spots” for catalytic activity and thus enhance the activity of nanozymes.

Unlike natural enzymes, the size and composition of most nanozymes are not uniform, with the exception of fullerene-based nanozymes. Further, batch-to-batch variation in size and shape of nanoparticles/nanozymes, and thus alterations in physicochemical properties, requires increased focus on improving the synthesis protocol in order to produce the monodispersed nanozymes with atomically precise structures. The rational design of an atomically precise nanozyme for a specific activity could be achieved by advanced computation-assisted technology. So far there are only limited types of enzymatic activities (SOD, peroxidase, catalase, and oxidase) which can be performed by nanozymes; therefore, nanozyme research needs to broaden more to cover other types of enzyme activities. Such efforts will help realize the clinical potential of nanozymes in nanomedicines, biotechnology, and other related areas.

Above all, the safety concerns of nanomaterials are currently receiving considerable attention due to their possible effects on human health and environment (Mahmoudi et al., 2011; Horie et al., 2012). “Safe-by-design” approach for nanomaterial/nanozyme synthesis could be utilized to develop biocompatible materials. Additionally, the coating of biocompatible polymers such as polyethylene glycol and dextran over the nanoparticles surface has been reported to impart biocompatibility. For example, dextran-coated iron oxide nanoparticles (Resovist) have been approved by the US Food and Drug Administration for clinical use. Therefore, more such nanozymes must be developed as a biocompatible catalyst for biomedical applications.

## Conclusion

The comprehensive summary of this review suggests that nanozymes are an emerging technology having the enormous potential of biomedical applications. Although the current literature of nanozymes has mostly covered the mimicking of four types of biological enzymes (SOD, catalase, oxidase, and peroxidase), nanozymes with other enzyme-like activities need to be synthesized. Antioxidant nanozymes have been shown to protect mammalian cells from the oxidative stress; however, pro-oxidant nanozymes are explored to use them in biosensing, and other immunoassays. Although most of the current nanozyme literature is about the i*n-vitro* catalytic activity, and immunoassay applications, however, few reports about their interaction at nano-bio interface. These studies are motivating but still leave many questions unanswered, which encourages further research. Detailed characterization of nanozymes upon administration *in vivo* conditions would shed light about the formation of protein corona and the interaction with cationic and anionic molecules dispersed in living organisms. Undoubtedly, in the coming years, research on nanozymes will continue to expand at the interface of nanomedicines, animal biotechnology, enzymology, and materials science.

## Author Contributions

The author confirms being the sole contributor of this work and has approved it for publication.

### Conflict of Interest Statement

The author declares that the research was conducted in the absence of any commercial or financial relationships that could be construed as a potential conflict of interest.
